# The ED_50_ and ED_95_ of Prophylactic Norepinephrine for Preventing Post-Spinal Hypotension During Cesarean Delivery Under Combined Spinal-Epidural Anesthesia: A Prospective Dose-Finding Study

**DOI:** 10.3389/fphar.2021.691809

**Published:** 2021-07-12

**Authors:** Wenping Xu, Dan Michael Drzymalski, Ling Ai, Hanqing Yao, Lin Liu, Fei Xiao

**Affiliations:** ^1^ Department of Anesthesia, Jiaxing University Affiliated Women and Children Hospital, Jiaxing, China; ^2^ Department of Anesthesiology and Perioperative Medicine, Tufts Medical Center, Boston, MA, United States

**Keywords:** cesarean delivery, anesthesia, spinal, norepinephrine, hypotension

## Abstract

**Background:** Hypotension commonly occurs with spinal anesthesia during cesarean delivery. Norepinephrine is an alternative to phenylephrine which can be used to prevent or treat hypotension, with better maintained cardiac output and less bradycardia. However, an appropriate initial prophylactic infusion dose of norepinephrine remains unclear. The aim of this study was to describe the dose-response relationship of prophylactic norepinephrine infusion during cesarean delivery under combined spinal-epidural anesthesia.

**Methods:** We performed a prospective, randomized, double-blinded dose-finding study. One hundred patients undergoing elective cesarean delivery were randomly assigned to receive an infusion of norepinephrine at 0, 0.025, 0.05, 0.075 or 0.1 μg/kg/min initiated immediately after intrathecal injection of 10 mg bupivacaine combined with 5 µg sufentanil. An effective dose was considered when there was no hypotension (systolic blood pressure < 90 mm Hg or < 80% of baseline) during the time period from injection of intrathecal local anesthetic to delivery of the neonate. The primary aim was to determine the dose-response relationship of norepinephrine to prevent spinal anesthesia-induced hypotension. The median effective dose (ED_50_) and 95% effective dose (ED_95_) for norepinephrine were calculated utilizing probit analysis.

**Results:** The proportion of patients with hypotension was 80, 70, 40, 15 and 5% at norepinephrine doses of 0, 0.025, 0.05, 0.075 and 0.1 μg/kg/min, respectively. The ED_50_ and ED_95_ were 0.042 (95% CI, 0.025–0.053) µg/kg/min and 0.097 (95% CI, 0.081–0.134) µg/kg/min, respectively. There were no differences in the Apgar scores (*p =* 0.685) or umbilical arterial pH (*p* = 0.485) measurements of the newborns among the treatment groups.

**Conclusion:** A norepinephrine infusion of 0.1 μg/kg/min as an initial starting dose was effective for the prevention of spinal-induced hypotension.

## Introduction

Hypotension frequently occurs during cesarean delivery (CD) after the initiation of spinal or combined spinal-epidural anesthesia (CSEA). Because hypotension increases maternal and neonatal morbidity, treating and/or preventing hypotension improves the quality of care provided by the obstetric anesthesiologist. While phenylephrine is commonly used in obstetric anesthesia practice, bradycardia and reduced cardiac output (CO) are considerable side effects associated with its use (NganKee, 2017a; Campbell and Stocks, 2018; Kinsella et al., 2018). Norepinephrine is an alternative vasopressor with significantly less bradycardia and better maintained cardiac output, thereby providing an attractive alternative to phenylephrine (NganKee, 2017a; NganKee, 2017b; Kinsella et al., 2018).

Besides choice of drug, the administration strategy for prophylactic vasopressor use is also important. While phenylephrine and norepinephrine are commonly administered as a bolus, recent literature suggests that administration as an infusion may be associated with less hypotension, nausea and vomiting (Allen et al., 2010; Heesen et al., 2014; Bishop et al., 2017; George et al., 2018). A prior study showed that prophylactic infusion of vasopressor resulted in significantly less severe post-spinal hypotension in both elective and urgent CD under spinal anesthesia (Bishop et al., 2017). Therefore, administration as an infusion appears to be favorable.

Although prophylactic infusion of norepinephrine has been reported, the appropriate initial starting infusion dose remains to be determined (Ahmed et al., 2019; Fu et al., 2020; Wei et al., 2020). Therefore, the primary aim of this study was to describe the dose-response relationship of an infusion of norepinephrine for the prevention of post-spinal hypotension between the time of the intrathecal local anesthetic injection to the time of neonatal delivery. The secondary aim was to investigate side effects, including those on maternal hemodynamics and neonatal outcomes.

## Materials and Methods

### Ethics

This study was approved by the Ethical Review Board of Jiaxing University Affiliated Women and Children Hospital, Jiaxing, China (No. 201900302). All patients enrolled in this study provided written informed consent. We registered the clinical trial in the Chinese Clinical Trial Registry (www.chictr.org, registration no. ChiCTRTRC1900022151, registration date: March 27, 2019. URL: http://www.chictr.org.cn/showproj.aspx?proj=37391) prior to patient enrollment. The first patient was enrolled on April 1, 2019.

### Enrollment

Patients were included in the study if they met the following inclusion criteria: a singleton pregnancy, scheduled for elective cesarean delivery. Exclusion criteria included: American Society of Anesthesiologists physical status classification > II, preeclampsia or coexistent hypertension (systolic blood pressure ≥ 140 mm Hg), preexisting or gestational diabetes (fasting blood-glucose > 125 mg/dl), body mass index > 35 kg/m^2^, extremes of height (less than 150 cm or more than 175 cm), and contraindications to neuraxial anesthesia. This clinical trial was performed with approval from the Department of Anesthesia, Jiaxing University Affiliated Women and Children Hospital.

### Randomization

A random number table was generated in Microsoft Excel (Redmond, Washington, United States ) and each assignment for every patient was secured in a sealed opaque, sequentially numbered envelope to prevent unblinding of observers. Patients were assigned randomly to one of five norepinephrine infusion groups: 0, 0.025, 0.05, 0.075 and 0.1 μg/kg/min. One of the investigators not involved in data collection, F. Xiao, prepared each infusion concentration in an unmarked 50 ml syringe (Lettered A, B, C, D or E) in advance to ensure blinding of the study solution. For each group, the dose of norepinephrine (in µg) was prepared using the following calculations: none for group 0, 1.5 × weight (kg) for group 0.025, 3.0 × weight (kg) for group 0.05, 4.5 × weight (kg) for group 0.075, and 6.0 × weight (kg) for group 0.1. Body weight was measured in the operating theater upon patient arrival to ensure accurate dosing.

### Study Procedures

Upon entry into the operating theater, each patient had an 18-gauge peripheral intravenous catheter placed, oxygen delivered via face mask at 3 L/min, and standard physiologic monitors applied (including non-invasive arterial blood pressure [NIBP] cuff, pulse oximeter and electrocardiography). Of note, the administration of premedication (e.g. midazolam) was not permitted per protocol.

With the patient in a left lateral decubitus position, a CSEA technique was performed by one of three anesthesiologists (YH Shen, L Liu, and WP Xu; all with more than 10 years of clinical experience) at the L3-4 vertebral interspace as determined by palpation. First, an 18-gauge Tuohy needle was used to access the epidural space with the loss-of-resistance to saline technique. Second, a 27-gauge Whitacre needle was passed through the Tuohy needle to reach the subarachnoid space. Once appearance of clear cerebrospinal fluid (CSF) was confirmed, 10 mg of hyperbaric bupivacaine with 5 µg of sufentanil were administrated intrathecally over approximately 20 s. The Whitacre needle was then withdrawn and an epidural catheter was inserted into the epidural space and gently aspirated to ensure there was no blood or CSF. Patients were then positioned supine with left uterine displacement using a 15° wedge. Immediately after the injection of the spinal anesthetic, administration of the study drug was started intravenously at a rate of 50 ml/h. Lactated Ringer’s solution warmed at 37°C was also administered, starting with a loading dose of 10 ml/kg given over 30 min.

### Data Gathering

For each patient, baseline systolic blood pressure (SBP) and heart rate (HR) were determined by calculating the mean of three separate measurements, assessed 3 min apart prior to the initiation of spinal anesthesia (Xiao et al., 2018; Lee et al., 2020). After intrathecal injection of local anesthetic, NIBP and HR were measured at 1 min intervals, and at 5 min intervals after delivery of the newborn. Hypotension was defined as a decrease of ≥ 20% of baseline SBP or an absolute SBP reading < 90 mm Hg, whichever occurred first. We defined hypertension as SBP > 120% of baseline value, and bradycardia as HR < 50 beats/min. Hypotension, bradycardia and hypertension were treated according to our institutional standards. For hypotension occurring with an increase in HR, 50 µg phenylephrine or 4 µg norepinephrine were given. For hypotension occurring with bradycardia, 0.5 mg atropine and/or 6 mg ephedrine were given. For hypertension occurring during the case, the study infusion was discontinued; the infusion was restarted when SBP returned to < 120% of baseline value. Norepinephrine infusion was determined to be effective when there was no single reading of hypotension from the time of spinal injection to delivery of the newborn. The study period was defined as the interval from injection of intrathecal local anesthetic to delivery of the neonate.

Sensory block level was assessed using an 18-gauge blunt needle at 5, 10, and 15 min after intrathecal local anesthetic administration. Patients were asked to describe if the blunt needle caused pain, felt sharp, or both caused pain and felt sharp. Surgery was permitted only if the sensory block level reached T6 or above, which was considered adequate when the patient stated the blunt needle no longer caused pain.

Demographic characteristics, including age, height, weight (as measured in the operating theater on a scale), and gestational age were recorded. Surgical data including duration of surgery and duration from induction of neuraxial anesthesia to newborn delivery were recorded. Physician interventions, defined as having to treat hypotension, hypertension, or bradycardia, as well as occurrence of side effects (hypotension, hypertension, bradycardia, nausea and vomiting, shivering), were recorded. Neonatal outcomes, including Apgar scores and umbilical arterial pH, were also recorded.

### Sample Size

Sample size was calculated with the Cochran–Armitage Test using PASS (version 11.0.7; NCSS, LLC, Kaysville, UT, United States). Calculations were based on internal preliminary data from an unpublished randomized pilot study, showing that for the five norepinephrine infusion groups of 0, 0.025, 0.05, 0.075 and 0.1 μg/kg/min, the proportions of hypotension were 75, 55, 40, 20 and 10%, respectively. We determined that a total sample size of 45 patients (at least nine patients per group) would be required to have 90% power to detect a linear trend among groups of patients with hypotension using a Z test with continuity correction and a significance level of 0.05. In order to account for potential patient attrition and to obtain narrower confidence intervals, we chose 20 patients for each group.

### Statistical Analysis

Distribution of univariable data was assessed using the Kolmogorov-Smirnov test. Normally distributed data were presented as mean ± standard deviation (SD) and analyzed using one-way analysis. The post hoc Bonferroni test was used for pairwise comparisons. Non-normally distributed data were presented as median (total range) and analyzed using the Kruskal-Wallis test. The post hoc Dunns test was applied to analyze pairwise comparisons. Categorical data such as proportion of patients with hypotension were analyzed using the Cochran-Armitage chi-square test for trend; if an overall test of difference among groups was significant, chi-square tests was used for pairwise comparison. Serial changes in SBP in the first 15 min after spinal anesthesia were analyzed using a summary measures technique. For each group, the area under the curve for values plotted against time was calculated using the trapezium rule, and these values were then compared between groups using one-way analysis of variance. The incidence and timing of hypotension were analyzed using Kaplan–Meier survival analysis with comparison between groups using the log-rank test. The ED_50_ and ED_95_ for an effective prophylactic norepinephrine infusion dose was determined using probit regression and 95% confidence intervals (CI) were reported. *p* < 0.05 was regarded as statistically significant (two-sided). Where Bonferroni corrections were applied, adjusted *P* values given by the software of GraphPad Prism are presented. Analyses were performed using IBM SPSS Statistics for Windows version 22.0 (IBM Corp, Armonk, NY) and GraphPad Prism version 5.0 (GraphPad Software Inc., San Diego, CA).

## Results

**FIGURE 1:**
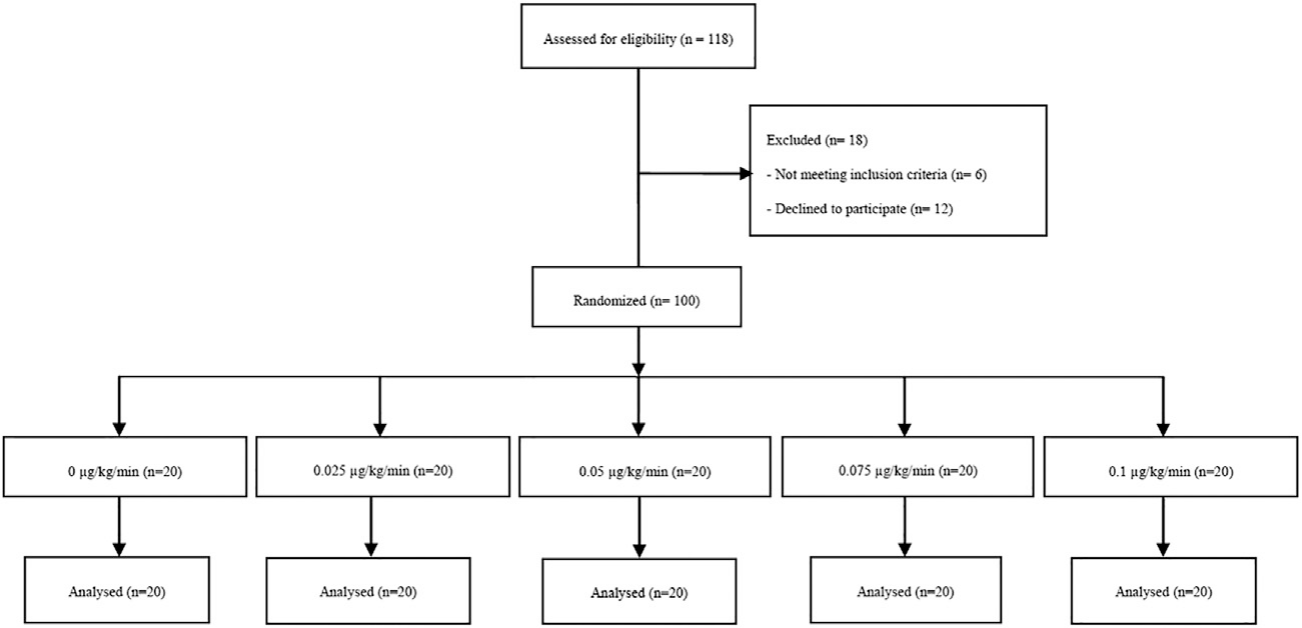
Consort Flow Diagram.

One hundred and eighteen patients scheduled for elective CD were assessed for eligibility. Twelve declined to participate and six did not meet inclusion criteria (Figure 1). No meaningful differences were found in the patient demographics, characteristics of spinal anesthesia, or surgical duration, suggesting adequate randomization (Table 1).

**TABLE 1:** Demographic data, surgical times and sensory block level.

	Group 0 (*n* = 20)	Group 0.025 (*n* = 20)	Group 0.05 (*n* = 20)	Group 0.075 (*n* = 20)	Group 0.1 (*n* = 20)
Age (years)	30 ± 3	30 ± 4	31 ± 5	31 ± 4	31 ± 4
Height (cm)	160 ± 6	160 ± 4	159 ± 5	160 ± 4	159 ± 5
Weight (kg)	70 ± 6	70 ± 6	71 ± 5	74 ± 8	73 ± 7
Gestational age (weeks)	39 ± 1	39 ± 1	38 ± 1	39 ± 1	39 ± 1
Spinal induction-delivery duration (min)	19.0 ± 2.6	19.3 ± 3.3	19.7 ± 3.1	19.1 ± 3.2	19.6 ± 3.0
Sensory block level (dermatome)	T4 (T2–T4)	T4 (T3–T4)	T4 (T3–T4)	T4 (T3–T5)	T4 (T2–T4)

Data are mean ± SD or median (range).

**FIGURE 2:**
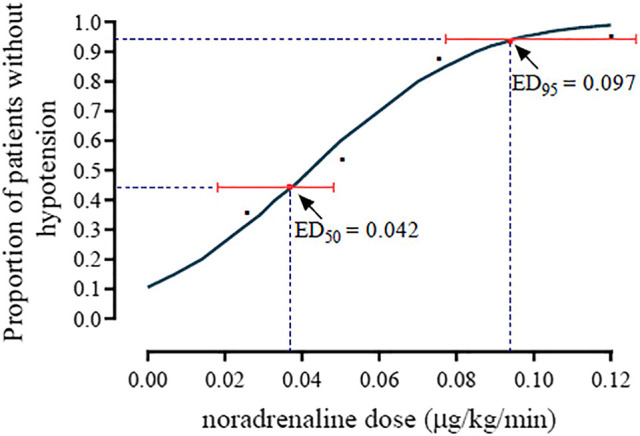
The dose-response curve of norepinephrine for preventing spinal anesthesia-induced hypotension. The ED50 and ED90 were 0.042 (95% CI, 0.025–0.053) µg/kg/min and 0.097 (95% CI, 0.081–0.134) µg/kg/min, respectively.

The incidence of hypotension was 80, 70, 40, 15 and 5% in the norepinephrine infusion groups of 0, 0.025, 0.05, 0.075 and 0.1 μg/kg/min, respectively. The dose-response curve of norepinephrine to prevent spinal anesthesia-induced hypotension is presented in Figure 2. The ED_50_ and ED_95_, which was determined from the probit analysis of hypotension incidence, were 0.042 (95% CI, 0.025–0.053) µg/kg/min, and 0.097 (95% CI, 0.081–0.134) µg/kg/min, respectively.

**FIGURE 3:**
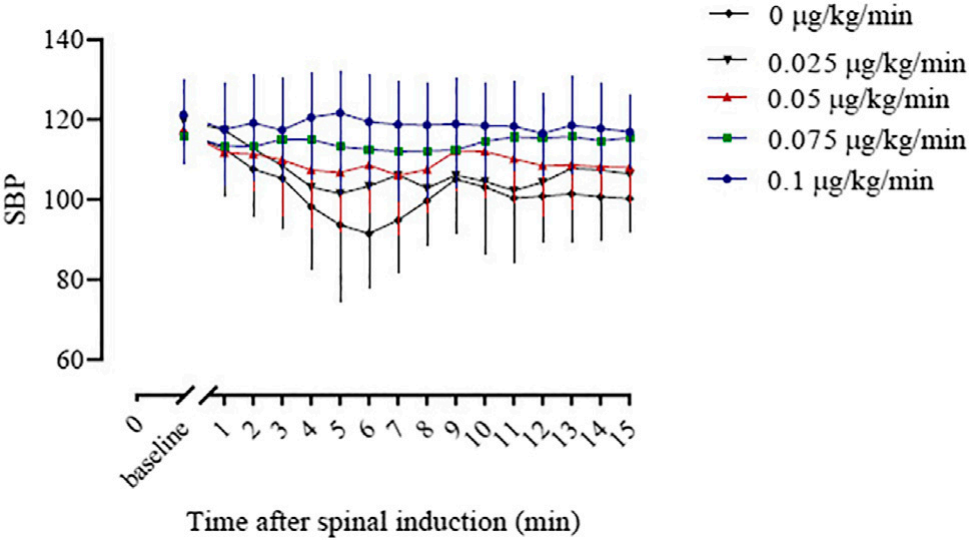
The baseline systolic blood pressure (SBP) and the SBP in the first 15 min after spinal induction is presented for the five groups. The area under the curve (mean ± SD) was significantly different among the groups (2,309 ± 33, 2,067 ± 34, 1,653 ± 25, 1,584 ± 31 and 1,572 ± 36 min × mmHg in groups 0, 0.025, 0.05, 0.075, and 0.1, respectively, *p* < 0.001).

The baseline SBP and the SBP in the first 15 min after induction of spinal anesthesia in the five groups are shown in Figure 3. The areas under the curve were 2,309 ± 33, 2,067 ± 34, 1,584 ± 31, 1,653 ± 25, and 1,464 ± 36 min × mm Hg in groups 0, 0.025, 0.05, 0.075 and 0.1 μg/kg/min, respectively, and there was a significant linear trend across groups (*p* < 0.001).

**FIGURE 4:**
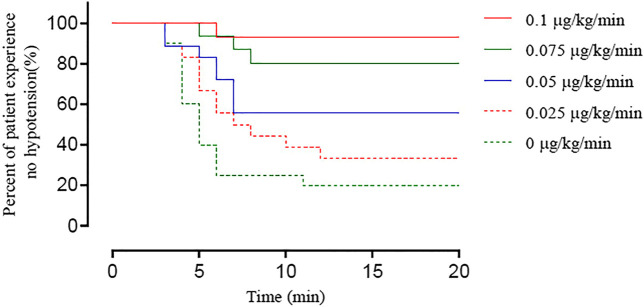
Kaplan–Meier survival curves showing the percentage of patients whose SBP remained > 80% of baseline or ≥ 90 mm Hg until newborn delivery. There was a significant difference among groups (log-rank test, *p* < 0.0001).

The timing and incidence of hypotension in the five groups using Kaplan-Meier curves are shown in Figure 4. The log-rank test showed a significant difference among groups (*p* < 0.001).

Side effects and neonatal outcomes are shown in Table 2. There was a significant difference in the incidence of hypotension among groups (Table 2, *p* < 0.0001). The incidence of hypotension was greater in groups with lower doses of norepinephrine, such that the incidence of hypotension in group 0 and 0.025 was significant higher than in group 0.075 and 0.1. No patients experienced hypertension or bradycardia. The incidence of nausea and vomiting was different among groups (*p* = 0.021). Other side-effects such as bradycardia and shivering did not differ among groups. No differences were found in the Apgar scores or umbilical arterial pH measurements among groups.

**TABLE 2:** Hemodynamic changes, side effects and neonatal outcome.

	Group 0 (*n* = 20)	Group 0.025 (*n* = 20)	Group 0.05 (*n* = 20)	Group 0.075 (*n* = 20)	Group 0.1 (*n* = 20)	*p*-value
Hypotension	16 (80)^a^	14 (70)^b^	8 (40)^c^	3 (15)	1 (5)	< 0.0001
Reactive hypertension	0	0	0	0	0	–
Bradycardia	0	0	0	0	0	–
Numbers of patients who required a physician intervention	16 (80)^a^	14 (70)^b^	8 (40)^c^	3 (15)	1 (5)	< 0.0001
Nausea or vomiting	8 (40)	4 (20)	3 (15)	3 (15)	2 (10)	0.022
Shivering	3 (15)	3 (15)	4 (20)	2 (10)	3 (15)	0.845
Apgar score	9 ± 1	9 ± 1	9 ± 1	9 ± 1	9 ± 1	0.685
Umbilical artery pH	7.27 ± 0.08	7.27 ± 0.08	7.28 ± 0.07	7.27 ± 0.08	7.29 ± 0.08	0.485

Data are presented as number (%), median (range) or mean ± SD. Categorical data were analyzed using the Cochran-Armitage chi-square test for trend. Reactive hypertension was defined as systolic blood pressure >120% of baseline value.

a *P* = 0.02 vs. group 0.05, *p* < 0.0001 vs. group 0.075 and 0.1.

b *P* = 0.001 vs. group 0.075, *p* < 0.0001 vs. group 0.1.

c *P* = 0.002, vs. group 0.1.

There were 16 (80%), 14 (70%), 8 (40%), 3 (15%) and 1 (5%) patients that required physician intervention in groups 0, 0.025, 0.05, 0.075 and 0.1 μg/kg/min, respectively, which was significantly different among groups (Table 2, *p* < 0.0001).

## Discussion

In this prospective, randomized, double-blinded study, we found that the ED_50_ and ED_95_ of prophylactic norepinephrine for the prevention of spinal hypotension were 0.042 (95% CI, 0.025–0.053) µg/kg/min, and 0.097 (95% CI, 0.081–0.134) µg/kg/min, respectively. Our data suggest that a dose of 0.1 μg/kg/min of norepinephrine, close to the ED_95_ value, may be an appropriate initial infusion dose to prevent spinal anesthesia-induced hypotension. However, titration may be necessary as not all patients will respond the same way. While the ED_90_ value of norepinephrine infusion under similar conditions has been previously described (Fu et al., 2020), our study expands on the literature by reporting the ED_95_ value and suggests that a higher starting dose of norepinephrine can be safely used.

The finding that an infusion dose of nearly 0.1 μg/kg/min of norepinephrine for the prevention of spinal-induced hypotension had acceptable results adds to the data published in our prior study. In the previous study (Wei et al., 2020) to determine the ED_50_ and ED_95_ of prophylactic norepinephrine, we found that 0.07 μg/kg/min of norepinephrine was an appropriate initial dose. However, in that prior study we studied a narrow dose range, from 0.04 to 0.07 μg/kg/min, so the present study expands on that data to a greater range. In the current study, our data suggests that 0.1 μg/kg/min norepinephrine produces a more stable SBP after spinal anesthesia. The incidence of hypotension with 0.07 μg/kg/min of norepinephrine was 15% in the prior study, higher than the 5% incidence of hypotension in the 0.1 μg/kg/min group in the present study. Therefore, 0.1 μg/kg/min norepinephrine is likely an appropriate initial, clinically practical, dose for the prevention of hypotension in cesarean delivery under spinal anesthesia.

After the introduction of phenylephrine as a vasopressor for obstetric anesthesia practice, norepinephrine was studied as an alternative (NganKee et al., 2015). The major benefits of norepinephrine are that it is pharmacologically characterized as a weak β-adrenergic agonist, with minimal bradycardia and better maintained cardiac output (CO), thereby promoting it as a clinical vasopressor to avoid spinal hypotension in cesarean delivery. *Hasanin et al.*(Ahmed et al., 2019) previously analyzed use of prophylactic norepinephrine infusion to prevent spinal hypotension previously and suggested 0.05 μg/kg/min as the appropriate dose. One possible reason for a lower dose compared to the present study might be the addition of a 5 μg norepinephrine bolus prior to initiating the norepinephrine infusion.

Fu et al. (2020) reported the ED_90_ value of norepinephrine to be 0.080 μg/kg/min after intrathecal injection of 15 mg hyperbaric ropivacaine, which was lower than that found in the present study but could be explained by differences in study protocols. However, even more interestingly, Fu et al. (2020) reported the incidence of post-spinal hypertension to be 35% with a norepinephrine infusion dose of 0.1 μg/kg/min while in our study we did not observe any hypertension. Possible explanations for these differences include a different target sensory block level (T5 vs. T4) and a different intrathecal local anesthetic (ropivacaine vs. bupivacaine) used between the two studies. Furthermore, the sample size of the current study was relatively small, which could account for some differences due to random chance. Future studies could examine the risk of reactive hypertension with norepinephrine infusion for cesarean delivery in more detail.

Titration of norepinephrine infusion has been recommended as it may result in more stable hemodynamics and less variability in SBP changes. Previously, Ngan Kee et al. (2018) conducted a study of varying norepinephrine infusion rates from 0 to 5 μg/min and the effects on post-spinal blood pressure. The authors observed a 17% rate of hypotension vs. 66% in the control group, comparable to the 15% rate in the 0.075 μg/kg/min group yet higher than the 5% incidence of hypotension in the 0.1 μg/kg/min group in the current study. However, the results do not clearly point towards one approach being more advantageous than the other. Further studies comparing titrated vs. weight-based fixed rates of vasopressor infusions are warranted. Taken together, our results provide valuable information that can inform initial rates for starting norepinephrine infusions for the prevention of post-spinal hypotension in cesarean delivery. Given the short duration of cesarean delivery in our institution, a fixed rate (0.1 μg/kg/min) appears to be efficient and safe.

When choosing phenylephrine vs. norepinephrine, an important consideration is the safety of administration via peripheral intravenous catheters. In our study, we did not observe any patients with peripheral ischemic complications. It is possible that because norepinephrine doses used in obstetric anesthesia are typically lower than those used for treating shock and because norepinephrine is used for a shorter duration, complications from its use would be less likely. However, future studies would need to be performed to definitively determine this to be the cause.

While our study has many strengths, its limitations should be acknowledged. First, while we have robust data regarding SBP and HR, assessment of CO would offer more detailed physiological data. However, due to the invasive nature of traditional CO monitoring methods, obtaining this data was not possible. Nevertheless, maternal HR is a reasonable surrogate for cardiac output because the stroke volume of healthy pregnant women has been shown to change minimally with spinal anesthesia (Kinsella et al., 2018). Second, because of the strict inclusion criteria, our results may not be generalizable to all cases. Patients whose height and weight fall outside of the range of those studied, or those who require emergency cesarean delivery, may require different doses. Finally, we only studied SBP from the time of intrathecal injection to newborn delivery. Post-delivery SBP management is also important, and may affect maternal outcomes. Further clinical studies should be performed to understand this better. Finally, due to the dose-response design, the incidence of hypotension was higher in the control and low-dose groups. To overcome this design shortcoming, the blood pressure was set to be measured at 1-min intervals to timely correct the possible hypotension related complications.

In conclusion, the ED_50_ and ED_95_ of prophylactic norepinephrine for the prevention of spinal hypotension were 0.042 (95% CI, 0.025–0.053) µg/kg/min, and 0.097 (95% CI, 0.081–0.134) µg/kg/min, respectively. Based on these findings, a norepinephrine infusion of 0.1 μg/kg/min may be an appropriate initial starting rate for the prevention of spinal-induced hypotension. Nevertheless, a fixed dose may not be appropriate for all patients and titration to achieve appropriate physiological conditions based on patient response should also be performed. Norepinephrine infusion is an effective strategy for the prevention of spinal-induced hypotension during cesarean delivery.

## Data Availability

The original contributions presented in the study are included in the article/supplementary material, further inquiries can be directed to the corresponding author.

## Abbreviations

ED50, median effective dose; ED95, 95% effective dose; CD, cesarean delivery; CSEA, combined spinal-epidural anesthesia; CO, cardiac output; NIBP, on-invasive arterial blood pressure; SBP, systolic blood pressure; HR, heart rate; CSF, cerebrospinal fluid; SD, standard deviation; CI, confidence intervals.
